# Serum aminoterminal type III procollagen peptide reflects increased vascular thickness in healthy, young adults

**DOI:** 10.1016/j.ijcha.2026.101876

**Published:** 2026-01-23

**Authors:** Manar Bitar, Dieter Samyn, Madeleine Helgesson, Martin Vink, Paul Pettersson-Pablo

**Affiliations:** a Department of Laboratory Medicine, Clinical Chemistry, Örebro University Hospital, Örebro, Sweden; b School of Medicine, Faculty of Medicine and Health, Örebro University, Örebro, Sweden

**Keywords:** Preclinical atherosclerosis, Extracellular matrix (ECM), Procollagen III, aminoterminal peptide (PIIINP), Carotid-intima media thickness (cIMT), Carotid-femoral pulse wave velocity (PWV)

## Abstract

**Background:**

Procollagen III, aminoterminal peptide (PIIINP) is a degradation product of collagen type III-synthesis. Collagen type III is distributed in many tissues, and an increase in serum PIIINP could reflect an increase in collagen turnover and pro-fibrotic activity. In this study, on a population of younger, healthy adults, we examined whether serum PIIINP correlates with early markers of vascular health, to evaluate its potential as a biomarker for early screening of preclinical cardiovascular risk.

**Methods:**

PIIINP levels, pulse wave velocity (PWV) and Carotid-intima media thickness (cIMT) was measured in 834 healthy, non-smoking, individuals aged 18–26. In univariable and multivariable linear regression models, we examined the association between PIIINP and vascular measurements, PWV and cIMT with adjustment for serum lipids, liver enzymes and systolic blood pressure.

**Results:**

The average of PIIINP, PWV and cIMT measurements in this population, were low (7.1 and 7.3 µg/L, 5.5 and 5.2 m/s, and 0.50 and 0.49 mm for men and women, respectively). In univariable analyses, PIIINP correlated positively with cIMT (p = 0.0061) and negatively with PWV (p = 0.0069). In multivariable analyses, a statistically significant association remained between PIIINP and cIMT (p < 0.001), but not with PWV.

**Conclusion:**

Serum PIIINP correlates with cIMT in a healthy population, indicating its potential as a biomarker of cardiovascular risk at a preclinical stage. PIIINP measurement being easier to perform and less examiner dependent than the more time consuming and cumbersome cIMT, are suggestive of its possible merits as an early screening tool for cardiovascular disease.

## 1 Introduction

The relationship between alterations in arterial extracellular matrix (ECM) and subclinical cardiovascular disease (CVD) has been the focus of rigorous research since the concept of vascular remodeling was first described [1]. Atherosclerosis is the progressive change in vascular structure and function that involves many components. Accumulation of collagen in ECM is one of the major contributing components of atherosclerosis plaques [2], [3] that increase arterial wall thickness and stiffness [4]. Collagen is mainly made up of collagen type I and III [5]. The gene for collagen type III is found to be significantly expressed in vulnerable plaques, indicating its involvement in the remodeling of atherosclerotic plaques [6]. Various biomarkers for monitoring ECM turnover have been proposed, of which the most common is the procollagen type III aminoterminal peptide (PIIINP) [7]. PIIINP is cleaved from procollagen III during collagen synthesis and released into circulation before the assembly of triple helix collagen fibrils, but some of it is also released upon collagen degradation [8]. Thus, the amount released increases in conjunction with an accelerated connective tissue turnover, which is a hallmark of fibrosis. PIIINP can be measured in serum, and since recently it has been done using a highly automated method [9].

Various clinical physiological measures can be used to assess the degree of endothelial dysfunction and vascular remodeling. Carotid-femoral pulse wave velocity (PWV) is a measurement of arterial stiffness [10], and carotid intima-media thickness (cIMT), is a measurement of structure alterations [11], [12], [13]. These measures are well-established as early markers for subclinical CVD and thus predictors of cardiovascular morbidity and mortality, independent of traditional risk factors [14] such as diabetes, hypertension, dyslipidemia, obesity and smoking.

The role of PIIIINP as a biomarker for ECM remodelling has been examined in hypertensive patients [15] before and after treatment [16], [17], and after myocardial infarction [18], [19]. Although PIIINP has been studied in patients with manifest vascular dysfunction and other morbidity [4], [12], [20], data on its potential role as a marker of early stages of CVD are scarce. In the present study we focus on healthy, young adults to examine whether PIIINP is associated also with the earliest stages of vascular dysfunction assessed by cIMT and PWV to evaluate its potential as a screening tool for early CVD.

## 2 Methods

Study population: Non-smokers, self-reported healthy; i.e, not diagnosed with any chronic disease, young Swedish adults were recruited at Örebro University and by advertisement in a local newspaper (n: 834, aged between 18 and 26). All study participants gave written consent before participation. Demographic-, lifestyle habit- and self-reported disease data was obtained via questionnaires on the same occasion as the measurement of blood pressure and blood sampling. The study has a cross-sectional design and was approved by the Uppsala Ethics Committee (Dnr: 2014/224).

PWV: Carotid-femoral pulse wave velocity was measured in all participants in a supine position after 25 min rest, using SphygmoCor (AtCor Medical Pty Ltd, SphygmoCor, Sydney, Australia), as previously described [22]. PWV is calculated by the formula: PWV (m/s) = distance between measurement locations (m)/transit time (s). The pulse wave propagation distance was obtained by subtraction of the length between sternal notch and carotid from the length between sternal notch and the femoral. Transit time was received after recording carotid-and femoral pulse waves using applanation tonometry with simultaneous electrocardiogram record. The difference in time between the top of pulse wave and the foot in the electrocardiogram for each of the carotid and femoral pulse waves was measured to obtain the transit time. Measurements of PWV were performed at least three times for each individual. A < 0,5 m/s difference between three measurements was considered to be acceptable, and the average of the three measurements was used in this study.

cIMT: The cIMT measurement was performed in all participants in a supine position with their heads slightly extended and turned approximately 45° to the left. Ultrasound measurement was carried out using a high-resolution ultrasound B-mode system, (GE Healthcare, Vivid E9, Chicago, Illinois, US) with a 12 MHz linear array transducer, on the common carotid artery, as has been previously described [23]. The measurements were repeated at least three times with an accepted difference < 0,05 mm, after which the average of the three maximum values was used in the study [24].

Systolic blood pressure (SBP): Blood pressure was measured after 15 min rest, in the left arm in supine position using a digital automated device (Dinamap V100; GE Healthcare, Buckinghamshire, UK). The measurement was repeated at least 3 times. The average of the last two SBP measurements between which the difference was less than 5 mmHg was reported [22].

PIIINP and other biomarkers: Venous blood samples were collected in a vacutainer tubes (BD Vacutainer; BD AB, Stockholm, Sweden) from all participants in a fasting state, after approximately 20 min rest. High sensitive C-reactive Protein (hs-CRP) was assayed by the Siemens High Sensitivity CRP Assay (ADVIA 1800 Chemistry System; Upplands Väsby, Sweden). The ADVIA Chemistry Alanine Aminotransferase (ALTP5P) and Aspartate Aminotransferase (ASTP5P)-methods were used to measure Alanine Aminotransferase (ALT) and Aspartate Aminotransferase (AST) on an ADVIA Chemistry XPT System. Direct cholesterol low density lipoprotein (direct LDL-Chol) was determined by a two-step colorimetric assay with VitrosMicroWell technology on a Vitros 5.1 system (Vitros 5.1TM FS, Clinical Chemistry Instruments, Raritan, NJ, USA). The ADVIA Centaur XPT platform was employed to analyse PIIINP with the ADVIA Centaur® −PIIINP assay, a two-site sandwich immunoassay using direct chemiluminometric technology. All laboratory analyses were performed in the accredited clinical chemistry laboratory at Örebro University Hospital.

Statistical analysis: All statistical calculations were performed with statistical program SPSS version 22 (IBM, Armonk, NY). To investigate whether the data was normally distributed, the mean and standard deviation (SD) where assessed, in addition to visual evaluation of histograms. In the case of abnormal distributed variables, the data was natural log (ln) transformed before inclusion in statistical calculations. Association between PIIINP and each of PWV and cIMT as dependent variables were performed by univariable and multivariable linear regression. In the multivariable models, LDL-Chol, hsCRP, AST, ALT, systolic blood pressure (SBP), sex and age were included as adjustment variables. Data for all variables were z-scored transformed, separately for each sex, before they were entered into the regression models. Missing values were due to participants dropping out before the second visit, or for technical reasons: hsCRP 5 participants, LDL-Chol 5 participants, PWV 13 participants, cIMT 10 participants, ALT 27 participants, AST 27 participants and PIIINP 28 participants. Associations between variables in different models were deemed to be statistically significant based on the 95% confidence intervals of the respective beta coefficient values, β.

## 3 Results

Participant characteristics are shown in Table 1. In this young population, cIMT and PWV means were on average low (0.50 and 0.49 mm, 5.5 and 5.2 m/s for men and women, respectively), as were the other cardiovascular risk factors, SBP (125 for men and 111 mmHg for women), LDL-cholesterol (2.30 and 2.31 mmol/L for men and women respectively) and CRP (0.84 mg/L for men and 1.11 mg/L for women), as expected in a population selected for being healthy. Some sex differences could be noted such as a slightly higher cIMT (p = 0.033) and PWV (p = <0.001) and lower PIIINP (7.1 µg/L in men and 7.3 µg/L in women, p = 0.498) among men.

**Table 1:** Participant characteristics.

		Men, n = 250 (Mean ± SD)		Women, n = 556 (Mean ± SD)
Age (years)		22.0 ± 2.0		21.8 ± 1.9
cIMT (mm)		0.504 ± 0.065		0.493 ± 0.057
PWV (m/s)		5.554 ± 0.845		5.223 ± 0.72
Systolic BP (mm Hg)		124.7 ± 11.9		110.8 ± 9.1
hsCRP (mg/L)		0.835 ± 0.862		1.111 ± 1.110
LDL-Chol (mmol/L)		2.303 ± 0.691		2.305 ± 0.707
AST (µkat/L)		0.410 ± 0.543		0.276 ± 0.152
ALT (µkat/L)		0.478 ± 0.307		0.352 ± 0.207
PIIINP (µg/L)		7.121 ± 2.168		7.322 ± 2.575

n: Number. SD: Standard deviation.

**Fig. 1:**
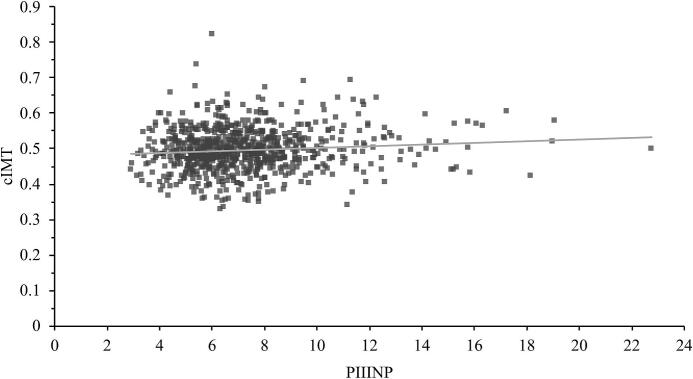
Scatter plot of PIIINP and cIMT.

**Fig. 2:**
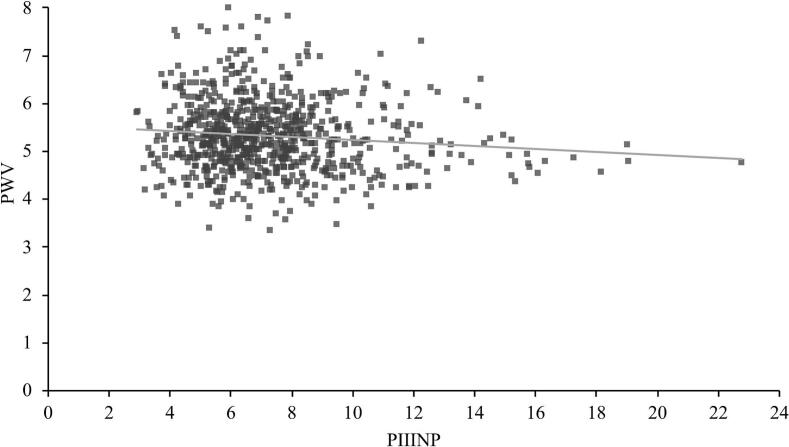
Scatter plot of PIIINP and PWV.

In univariable linear regression, PIIINP was statistically significantly positively correlated with cIMT, and negatively correlated with PWV (Table 2). Scatter plots showing the distribution of the results are shown in Fig. 1, Fig. 2. The slope of the curves are flat with associations between PIIINP and vascular biomarkers being small, as shown by generally low standardized beta coefficients, of 0.097 for cIMT (p = 0.0061) and −0.096 for PWV (p = 0.0069) (Table 2). In both univariate models, the coefficient of determination, R^2^, was low (0.0081 and 0.0084).

**Table 2:** Association of cIMT and PWV with PIIINP alone (univariable), in combination with age and sex in model 1, and in combination with age, sex, LDL-Chol, SBP, AST, ALT and hsCRP in model 2.

**cIMT**						
		β_PIIINP_ (95% CI)		p		R^2^ adj
Univariable		0.097 (0.027 – 0.166)		0.0061		0.0081
Multivariable, model 1		0.118 (0.048 – 0.189)		< 0.001		0.019
Multivariable, model 2		0.119 (0.049 – 0.188)		< 0.001		0.050
**PWV**						
		β_PIIINP_ (95% CI)		p		R^2^ adj
Univariable		−0.096 (−0.165 – −0.027)		0.0069		0.0084
Multivariable, model 1		−0.071 (−0.140 – −0.001)		0.048		0.024
Multivariable, model 2		−0.064 (−0.130 – 0.003)		0.062		0.121

cIMT: Carotid intima-media thickness. PWV: pulse wave velocity. β_PIIINP:_ standardized β coefficient for PIIINP. CI: Confidence interval. Model 1 includes PIIINP, age and sex. Model 2 includes PIIINP, LDL-Chol, SBP, hsCRP, AST, ALT, sex and age.

In multivariable linear regression, the associations between PIIINP and each of cIMT and PWV were analyzed in two steps: In the first step, Model 1, we adjusted for age and sex. As the age range of the population was small (18–26 year) with negligible differences in measurement variables between sexes, the adjustment in Model 1 (Table 2) had only minor impact on the associations observed in the univariable analyses. To control for the effect of serum lipids, blood pressure, inflammation and any possible liver related anomaly on either vascular biomarkers or PIIINP, adjustment for age, sex, SBP, hsCRP, AST, ALT and LDL-Chol was made in Model 2 (Table 2). For cIMT, the association with PIIINP remained statistically significant after adjustment in Model 1 and 2, with beta coefficients increasing slightly to 0.119 (p < 0.001) in Model 2. For PWV, the association with PIIINP remained statistically significant after adjustment in model 1 (p = 0.048) but further adjustment in Model 2 resulted in a less negative beta coefficient (from −0.096 to −0.064) and the association was no longer statistically significant (p = 0.062). R^2^ increased in both of the adjusted models, from 0.0081 to 0.050 for cIMT and from 0.0084 to 0.12 for PWV.

## 4 Discussion

In this study, we examined the association between PIIINP, as a biomarker for collagen type III turnover, and two surrogate markers for subclinical cardiovascular disease, cIMT and PWV, in a cohort of young, healthy adults. A statistically significant positive association was found between PIIINP and cIMT, while a negative association was found for PWV, that did not remain statistically significant in the second multivariable model. As PIIINP is well-established as a biomarker for liver fibrosis, adjustment of the regression models was made with AST and ALT, as well as other possibly confounding factors, but this did not impact the statistically significant association of PIIINP with cIMT.

The ECM remodeling process is step-wise, in which elastin in healthy vessels are replaced by proteoglycans in the early atherosclerotic lesions, which subsequently are replaced by collagen [7]. Collagen has been found to constitute 60% of the total plaque protein in advanced atherosclerotic lesions [25]. In our study’s young population, no plaques were observed in the carotid arteries, and cIMT measurements were on average low, so any increases would correspond only to the earliest stages of an elevated collagen III turnover. In a previous study, the Framingham offspring study [26], higher PIIINP concentrations showed a borderline association with carotid stenosis but not with cIMT. This differs from our findings. An increased cIMT is a subclinical phenomenon, while a stenosis denotes an overt dysfunction in compensating mechanisms of the affected vessels. The results of our study are more in concordance with those by Agarwal et al [21], who observed an association between PIIINP and cIMT on older patients. This association being found in a young, healthy population, indicates that PIIINP increases at early stages, being sensitive enough to detect even preclinical vascular remodeling.

In our study, PIIINP correlated inversely with PWV in a univariable model. This was unexpected as PWV and cIMT are both surrogate measures of vascular health and normally correlate. After adjustment for established CVD risk factors, the association between PWV and PIIINP was no longer significant. It is likely that PIIINP is also sensitive as a marker to non-fibrosis related factors, such as liver affection or systemic inflammation, and it is possible that this played a role in our results, given the differing results in Model 2, with a large increase in R^2^, when we adjusted for such factors. In this population, mean values of PWV, PIIINP, and cIMT were low, with measurement results below cutoffs established by the European Society of Cardiology (ESC); (PWV over 10 m/s or cIMT over 0.9 mm) [9], [27], [28] (Table 1). The low variance in measurement results, with pathological results with respect to established clinical cutoffs being rare, may have had an impact on the statistical models. The generally low coefficient of determinations, R2, in our linear regression models (Table 2) and the large spread of results in the scatter plots in Fig. 1, Fig. 2 are also indicative of this. In multivariable models, the adjustments rendered the association between PWV and PIIINP non-significant, while it strengthened the relationship with cIMT (from a beta coefficient of 0.097 to 0.119), which indicate a more robust relationship between cIMT and PIIINP. Furthermore, it is possible that temporality play a role in the negative univariable relationship between PWV and PIIINP. An increased PWV can be more dynamic, sometimes reversible and can arise at a different stage, possibly later in the pathogenetic process than cIMT, in some individuals. In a study of the association between PIIINP and PWV, on women with hypertension [4], it was observed that PIIINP was significantly associated with PWV only in those with a SBP higher than 160 mmHg but not in those with less than 160 mmHg. It was hypothesized that this could be explained by an accelerated development of arterial stiffness in those with higher SBP. Such results could be part of an explanation as to why PIIINP showed a negative, rather than positive, correlation with PWV in our normotensive population.

There are limitations of this study. The cross-sectional design, which did not permit inferences of causality in the associations found, is a limitation. It prevents determining the order in which the biomarkers, especially cIMT and PWV, increase in the subjects who showed elevated results. Furthermore, we studied only one marker for collagen turnover, chosen for lending itself well to high throughput analysis in a clinical setting. PIIINP is released by many tissues and can increase both due to a fibrotic or other collagen turnover process, in a non-vascular organ system. The selection of only healthy, young individuals and our adjustment variables in the multivariable models, was made to minimize this risk. Future studies employing a longitudinal design are warranted to examine the predictive capacity of PIIINP in relation to clinical outcomes. The strengths of our study were the large sample size that allowed the investigation of two different surrogate measurements that are comparatively little studied in this age group.

**In conclusion**, serum PIIINP was significantly, positively associated with cIMT and negatively with PWV but in multivariable regression models, adjusting for variables that could influence either PIIINP or the vascular measurements, the significant association with PWV did not remain. The positive correlation between PIIINP and cIMT, that was detectable in a young, healthy population, makes PIIINP an interesting candidate as an early screening tool for arterial disease. The highly automated, easy to use, commercial assay used in this paper, holds merit for screening larger populations compared to more time-consuming and examiner dependent methods such as cIMT.

## CRediT authorship contribution statement

**Manar Bitar:** Writing – original draft, Investigation, Data curation. **Dieter Samyn:** Writing – review & editing, Data curation. **Madeleine Helgesson:** Investigation, Data curation. **Martin Vink:** Writing – review & editing, Supervision. **Paul Pettersson-Pablo:** Writing – review & editing, Supervision, Methodology, Investigation, Conceptualization.

## Funding

Funding was provided by Region Örebro County’s Research Committee, Örebro, Sweden [OLL-780061] and Asset Management Arm (AFA) life insurance nr: 130275. The funding bodies had no influence on study design, data collection, analysis of data, or the manuscript.

## Declaration of competing interest

The authors declare that they have no known competing financial interests or personal relationships that could have appeared to influence the work reported in this paper.
